# Laguerre Wavelet Approach for a Two-Dimensional Time–Space Fractional Schrödinger Equation

**DOI:** 10.3390/e24081105

**Published:** 2022-08-11

**Authors:** Stelios Bekiros, Samaneh Soradi-Zeid, Jun Mou, Amin Yousefpour, Ernesto Zambrano-Serrano, Hadi Jahanshahi

**Affiliations:** 1 FEMA, University of Malta, MSD 2080 Msida, Malta; 2 LSE Health, Department of Health Policy, London School of Economics and Political Science, London WC2A 2AE, UK; 3 IPAG Business School (IPAG), 184, bd Saint-Germain, 75006 Paris, France; 4 Faculty of Industry and Mining (Khash), University of Sistan and Baluchestan, Zahedan 9816745845, Iran; 5 School of Mechanical Engineering and Automation, Dalian Polytechnic University, Dalian 116034, China; 6 Department of Mechanical and Aerospace Engineering, University of California, Irvine, CA 94720, USA; 7 Facultad de Ingeniería Mecánica y Eléctrica, Universidad Autónoma de Nuevo León, Av. Universidad S/N, Cd. Universitaria, San Nicolás de los Garza C.P. 66455, NL, Mexico; 8 Department of Mechanical Engineering, University of Manitoba, Winnipeg, MB R3T 5V6, Canada or

**Keywords:** Schrödinger equation, two-dimensional fractional equation, fractional derivative, Laguerre wavelet

## Abstract

This article is devoted to the determination of numerical solutions for the two-dimensional time–spacefractional Schrödinger equation. To do this, the unknown parameters are obtained using the Laguerre wavelet approach. We discretize the problem by using this technique. Then, we solve the discretized nonlinear problem by means of a collocation method. The method was proven to give very accurate results. The given numerical examples support this claim.

## 1. Introduction

In the 19th century, Reimann and Liouville as well as Euler and Fourier provided useful results in modern calculus and introduced the first foundational contributions in fractional calculus. Since the beginning of the 21st century, the subject of fractional calculus has been given a lot of attention by many researchers because of its more precise and realistic results in many industrial and technological fields. Today, it is known that many physical processes in nature can be modelled by using fractional calculus in a better way. The capacity of fractional calculus to meet the demands of the real-world applications provides a basis for the application of fractional modelling and fractional systems in the fields of engineering and applied science [1,2,3].

This ever-increasing expansion of fractional concepts has also made a significant contribution to the investigation of different fractional differential contexts [4,5]. The reviewed articles in [6] provide an up-to-date bibliography of approximate methods for the resolution of the fractional differential equations.

Beyond the great interest aroused by fractional equations, the fractional Schrödinger equation is one of the most important models in mathematical physics, and it has been used widely in various fields such as super-fluids, quantum mechanics, particle physics and plasma, earthquake science, nonlinear optics, underwater acoustics, the semi-conductor industry, and optimal control problems [7,8,9]. To the best of our knowledge, the existence of solutions for the above-mentioned equation has been studied in [10,11,12,13]. Although many researchers have provided efficient computational methods to solve the fractional Schrödinger equation, most of them are studied in one dimension. Indeed, to the best of our knowledge, only a limited number of methods has been generalized in order to deal with multi-dimensional time–space fractional Schrödinger equations [14,15,16].

Our goal in this paper is to present an efficient, high-accuracy, and simple method for solving the two-dimensional time–space fractional Schrödinger equation, which is defined as follows:

(1) $$i{\;}_{0}^{C}D_{t}^{\alpha }U+\kappa_{1}{\;}_{0}^{C}D_{x}^{\beta }U+\kappa_{2}{\;}_{0}^{C}D_{y}^{\beta }U+G\left(x,y,t\right)U=0,\;\;\;\left(x,y,t\right)\in \Omega \times \left[0,T\right],$$

with the initial condition

U(x,y,0)=ϕ(x,y),(x,y)∈Ω

and boundary condition

U(x,y,t)=0,(x,y)∈∂Ω.

In Equation (1), 0<α≤1 and 1/2<β≤2 are the order of Caputo fractional derivatives in time and space, respectively. Moreover, Ω is a bounded domain in $R^{2}$, $\kappa_{1}$ and $\kappa_{2}$ are real constants, ϕ(x,y) is a sufficient smooth function, *i* is the imaginary unity, and U(x,y,t) is assumed to be a complex wave function of space and time. In this paper, we propose a high-accuracy numerical method in both time and space to obtain the approximate numerical solutions for Equation (1). More precisely, for the proposed method, the Laguerre wavelets are considered as an expansion of the series. Due to the high accuracy and ease of use of these polynomials, researchers often apply them to find approximate solutions for a variety of problems. To simplify the problem significantly, Equation (1) is equivalently reformulated into a system of discrete equations. The unknown coefficients are chosen to satisfy the solution of Equation (1).

The rest of this work is organized as follows. The basic concepts of fractional operations are introduced in Section 2. In addition, some of the main characteristics of the Laguerre polynomials and wavelets are given in this section. The discretization and description of the method are explained in Section 3. Section 4 describes the approximate solutions obtained by the proposed method. Finally, the conclusion is given in the last section.

## 2. Preliminary Tools

Here, we provide some definitions and basic concepts that are used throughout the paper.

### 2.1. Fractional Operations

First, we briefly introduce some definitions related to fractional operators that permit us to formulate the two-dimensional time–space fractional Schrödinger Equation (1). For more details, see [17,18,19].

**Definition** **1.** *For a given function $f\in L_{1}\left(\left[t_{0},t_{f}\right],R^{n}\right)$, the α-order (α>0) of the Riemann–Liouville fractional integral is given by*

(2) $${}_{t_{0}}I_{t}^{\alpha }f\left(t\right)=\frac{1}{\Gamma (\alpha )}\int_{t_{0}}^{t}{\left(t-\tau \right)}^{\alpha -1}f\left(\tau \right)d\tau ,$$

*in which Γ(.) is the Euler Gamma function. It is clear that when α=0 then ${}_{t_{0}}I_{t}^{\alpha }f\left(t\right)=f\left(t\right)$.*

**Definition** **2.** *The 0<α-order of a fractional derivative for a given function f(t) in the Riemann–Liouville sense is determined by*

(3) $${}_{t_{0}}D_{t}^{\alpha }f\left(t\right)=D^{n}\left({\;}_{t_{0}}I_{t}^{n-\alpha }\right)f\left(t\right)=\frac{1}{\Gamma (n-\alpha )}{\left(\frac{d}{dt}\right)}^{n}\int_{t_{0}}^{t}{\left(t-\tau \right)}^{n-\alpha -1}f\left(\tau \right)d\tau ,$$

*where n−1<α≤n, n∈N. If α∈N, then the definition of the fractional derivative coincides with that of the ordinary derivative.*

**Definition** **3.** *The 0<α-order of Caputo fractional derivatives for a given function f(t) is specified as*

(4) $${}_{t_{0}}^{C}D_{t}^{\alpha }f\left(t\right)=\left({\;}_{t_{0}}I_{t}^{n-\alpha }\right)D^{n}f\left(t\right)=\frac{1}{\Gamma (n-\alpha )}\int_{t_{0}}^{t}{\left(t-\tau \right)}^{n-\alpha -1}f^{\left(n\right)}\left(\tau \right)d\tau ,$$

*in which n−1<α≤n, n∈N.*

Furthermore, if *K* is constant, then we have

$${}_{t_{0}}^{C}D_{t}^{\alpha }K=0.$$

In addition, the fractional derivative of f(t)=t for n−1<α≤n is obtained, as follows [20]:

$$\begin{array}{c} {}_{t_{0}}^{C}D_{t}^{\alpha }\left(t-t_{0}\right)=\left\{\begin{array}{c} \frac{\Gamma (n+1)}{\Gamma (n-\alpha +1)}{\left(t-t_{0}\right)}^{n-\alpha },\;n\geq \left[\alpha \right],\\ 0,\;\;\;\;\;\;\;n<[\alpha ], \end{array}\right. \end{array}$$

in which [α] is the integer part of α.

**Remark** **1.** *Let t>0, n−1<α<n, n∈R. If both functions, f(t) and g(t), together with their derivatives are continuous in [0,t], then the following Leibniz rule is valid for the Caputo derivative:*

$${}_{0}^{C}D_{t}^{\alpha }\left(f\left(t\right)g\left(t\right)\right)=\sum_{k=0}^{\infty }C_{\alpha }^{k}{(}_{0}D_{t}^{\alpha -n}f\left(t\right))g^{\left(k\right)}\left(t\right)-\sum_{k=0}^{n-1}\frac{t^{k-\alpha }}{\Gamma (k+1-\alpha )}\left({\left(f\left(t\right)g\left(t\right)\right)}^{k}\left(0\right)\right),$$

*wherein $C_{\alpha }^{k}=\left(\begin{array}{c} \alpha \\ k \end{array}\right)$.*

### 2.2. Laguerre Polynomials and Laguerre Wavelets

For any σ>−1, the Laguerre polynomials $L_{k}^{\left(\sigma \right)}\left(x\right)$, k≥0 are the eigenfunctions of the singular Sturm–Liouville problem in (0,∞), which are expressed as:

$${\left(x^{\sigma +1}exp\left(-x\right){\left(L_{n}^{\left(\sigma \right)}\left(x\right)\right)}^{{}^{\prime }}\right)}^{{}^{\prime }}+nx^{\sigma }exp\left(-x\right)L_{n}^{\left(\sigma \right)}\left(x\right)=0.$$

These polynomials are orthogonal with respect to the weight $w\left(x\right)=x^{\sigma }exp\left(-x\right)$ in (0,∞), that is

$$\int_{0}^{\infty }L_{n}^{\left(\sigma \right)}\left(x\right)L_{m}^{\left(\sigma \right)}\left(x\right)x^{\sigma }exp\left(-x\right)dx=\frac{(n+\sigma )!}{n!}\delta_{nm},\;\;\;n,m\geq 0,$$

where $\delta_{nm}$ is the *Dirac* delta function [21,22]. Furthermore, the following recursion relation is credible for the Laguerre polynomial:

$$L_{n+1}^{\left(\sigma \right)}\left(x\right)=\frac{\left(2n+\sigma +1-x\right)}{n+1}L_{n}^{\left(\sigma \right)}\left(x\right)-\frac{\left(n+\sigma \right)}{n+1}L_{n-1}^{\left(\sigma \right)}\left(x\right),$$

in which $L_{0}^{\left(\sigma \right)}\left(x\right)=1$ and $L_{1}^{\left(\sigma \right)}\left(x\right)=\sigma +1-x$. Moreover, Rodriguez’s formula in this case becomes:

$$L_{n}^{\left(\sigma \right)}\left(x\right)=\frac{t^{-\sigma }exp\left(x\right)}{n!}\frac{d^{n}}{dx^{n}}\left(x^{n+\sigma }exp\left(-x\right)\right).$$

Let $L_{n}\left(x\right)=L_{n}^{\left(0\right)}\left(x\right)$ for the special case σ=0. Then, we have $L_{n}^{\left(0\right)}\left(0\right)=1$, have caused these polynomials to become orthonormal in (0,∞). We recall a well-known classical global uniform for Laguerre polynomials, estimated as follows [21]:

$$|L_{n}^{\left(\sigma \right)}\left(x\right)|\leq \frac{(\sigma +1)k}{k!}exp\left(\frac{x}{2}\right),\;\sigma \geq 0,\;\;x\geq 0,\;\;k=0,1,2,\cdots .$$

The Laguerre wavelets in interval [0,1) are defined as follows:

(5) $$\begin{array}{c} \psi_{n,m}\left(x\right)=\left\{\begin{array}{c} \frac{2^{\frac{k}{2}}}{m!}L_{m}\left(2^{k}x-2n+1\right),\;\frac{n-1}{2^{k-1}}\leq x<\frac{n}{2^{k-1}},\\ 0,\;\;\;\;\;\mathrm{otherwhere}, \end{array}\right. \end{array}$$

for $n=1,2,3,\cdots ,2^{k-1}$, $k\in Z^{+}$. In addition, the order of Laguerre polynomials is denoted by m=0,1,⋯,M−1, in which *M* is a constant positive integer [23]. In addition, two-dimensional Laguerre wavelets are introduced as follows:

(6) $$\begin{array}{c} \psi_{n,m,l,j}\left(x,y\right)=\left\{\begin{array}{c} \frac{2^{\frac{k_{1}+k_{2}}{2}}}{m!j!}L_{m}\left(2^{k_{1}}x-2n+1\right)L_{j}\left(2^{k_{2}}y-2n+1\right),\;\;\frac{n-1}{2^{k_{1}-1}}\leq x<\frac{n}{2^{k_{1}-1}},\frac{l-1}{2^{k_{2}-1}}\leq y<\frac{l}{2^{k_{2}-1}},\\ 0,\;\;\;\;\;\;\;\mathrm{otherwhere}, \end{array}\right. \end{array}$$

in which $l=1,2,\cdots ,2^{k_{2}-1}$, $n=1,2,\cdots ,2^{k_{1}-1}$, $k_{1}$, and $k_{2}$ are arbitrary positive integers. Furthermore, the order of Laguerre polynomials in relation (6) are indicated by *m* and *j*.

## 3. Proposed Computational Method by Laguerre Wavelets

A given function f(x)∈C[0,1] can be expanded in terms of Laguerre wavelets in the following form:

(7) $$f\left(x\right)=\sum_{n=1}^{\infty }\sum_{m=0}^{\infty }c_{nm}\psi_{nm}\left(x\right),$$

where $c_{nm}=<f\left(x\right),\psi_{nm}\left(x\right)>$, <.,.> denotes the inner product in $L^{2}\left[0,1\right]$, and $\psi_{nm}\left(x\right)$ are the Laguerre wavelets defined in Equation (5). If the infinite series in Equation (7) are truncated, then we can rewrite it as follows:

(8) $$f\left(x\right)≅\sum_{n=1}^{2^{k-1}}\sum_{m=0}^{M-1}c_{nm}\psi_{nm}\left(x\right)=C^{T}\Psi \left(x\right),$$

in which Ψ(x) and *C* are two $2^{k-1}M$ column vectors and are given respectively by

$$\Psi \left(x\right)=[\psi_{1,0}\left(x\right),\psi_{1,1}\left(x\right),\cdots ,\psi_{1,(M-1)}\left(x\right),\cdots ,\psi_{2^{k-1},\left(M-1\right)}\left(x\right)],$$

and

$$C={\left[c_{1,0},c_{1,1},\cdots ,c_{1,(M-1)},c_{20},\cdots ,c_{2^{k-1},\left(M-1\right)}\right]}^{T},$$

wherein

$$c_{nm}=\int_{0}^{1}\psi_{nm}\left(x\right)f\left(x\right)dx.$$

Furthermore, a function f(x,y) over [0,1)×[0,1) can be expanded in terms of two-dimensional Laguerre wavelets, as follows:

(9) $$f\left(x,y\right)≅\sum_{n=1}^{2^{k_{1}-1}}\sum_{m=0}^{M_{1}-1}\sum_{l=1}^{2^{k_{2}-1}}\sum_{j=0}^{M_{2}-1}c_{nmlj}\psi_{nmlj}\left(x,y\right)=C^{T}\Psi \left(x,y\right),$$

in which Ψ and *C* are two $2^{k_{1}-1}2^{k_{2}-1}M_{1}M_{2}\times 1$-dimensional matrix whose elements can be calculated by

$$\begin{array}{cc} \Psi (x,y)= & [\Psi_{1,0,1,0}\left(x,y\right),\Psi_{1,0,1,1}\left(x,y\right),\ldots ,\Psi_{1,0,1,(M_{2}-1)}\left(x,y\right),\\ & \;\ldots ,\Psi_{1,0,2^{k_{2}-1},\left(M_{2}-1\right)}\left(x\right),\ldots ,\Psi_{2^{k_{1}-1},\left(M_{1}-1\right),2^{k_{2}-1},\left(M_{2}-1\right)}], \end{array}$$

and

$$c_{nmlj}=\int_{0}^{1}\int_{0}^{1}\psi_{nm}\left(x\right)\psi_{lj}\left(y\right)f\left(x,y\right)dxdy,$$

where the coefficients $c_{nmlj}$ are known. In order to solve problem (1), and paying attention to the definition of Laguerre wavelets in (5), we first consider the collocation points $t_{l}=\frac{l}{T^{*}-1}$, $l=0,1,\cdots ,T^{*}-1$ in which $T^{*}=2^{k-1}\left(M-1\right)$. Hence, we can get the following Laguerre wavelet matrix:

(10) $$L_{T^{*}\times T^{*}}=\left[\Psi \left(0\right),\Psi \left(\frac{1}{T^{*}-1}\right),\cdots ,\Psi \left(1\right)\right].$$

Furthermore, the fractional derivative of the Laguerre polynomials is obtained as follows [24]:

(11) $${}_{0}^{C}D_{t}^{\alpha }L_{n}\left(t\right)=\sum_{k=[\alpha ]}^{n}\frac{{\left(-1\right)}^{k}}{k!}\frac{n!}{k!(n-k)!}\frac{t^{k-\alpha }}{\Gamma (k-\alpha +1)}.$$

Therefore, for the fractional derivative of Ψ(t), we have

(12) $$\left(D^{\alpha }\Psi \right)\left(t\right)≅\left(L_{T^{*}\times T^{*}}S^{\alpha }L_{T^{*}\times T^{*}}^{-1}\right)\Psi \left(t\right),$$

where $S^{\alpha }$ is defined as follows:

ST*×T*α=1(T*−1)αΓ(α+2)0s1,110s1,2+20s2,2…∑j=1T*j0sj,T*0−s1,1−[11s1,2+21s2,2]…−∑j=2T*j1sj,T*0022s2,2…∑j=3T*j2sj,T*⋮⋮⋮⋱⋮000⋯(−1)T*T*T*sT*,T*

in which

$$s_{j,k}=\left\{\begin{array}{c} {\left(-1\right)}^{j}\frac{j!}{\Gamma (j+1-\alpha )}\left(\begin{array}{c} k\\ j \end{array}\right),\;\;\mathrm{if}\;\;k\geq j\geq \left\lceil \alpha \right\rceil \\ 0,\;\;\;\;\;\mathrm{otherwise}. \end{array}\right.$$

By using the above collocation points $t_{l}$, we first expand $U(x,y,t_{l})$ using the two-dimensional extension of the Laguerre wavelets (9). Then, Equation (12) is used to approximate the fractional derivatives of $U(x,y,t_{l})$. By applying these approximations, we obtain the discretization form of (1) as follows:

(13) $$i{\;}_{0}^{C}D_{t}^{\alpha }U_{k,M}\left(x,y\right)+\kappa_{1}{\;}_{0}^{C}D_{x}^{\beta }U_{k,M}\left(x,y\right)+\kappa_{2}{\;}_{0}^{C}D_{y}^{\beta }U_{k,M}\left(x,y\right)+G_{k,M}\left(x,y\right)U_{k,M}\left(x,y\right)=0.$$

Let $\left(x_{m},y_{j}\right)$ be the set of $2^{k_{1}-1}M_{1}\times 2^{k_{2}-1}M_{2}$ zeros of the Laguerre polynomials in [0,1]. Then, we can collocate Equation (13) in the following form:

(14) $$i{\;}_{0}^{C}D_{t}^{\alpha }U_{k,M}\left(x_{m},y_{j}\right)+\kappa_{1}{\;}_{0}^{C}D_{x}^{\beta }U_{k,M}\left(x_{m},y_{j}\right)+\kappa_{2}{\;}_{0}^{C}D_{y}^{\beta }U_{k,M}\left(x_{m},y_{j}\right)+G_{k,M}\left(x_{m},y_{j}\right)U_{k,M}\left(x_{m},y_{j}\right)=0.$$

Finally, Equation (14) can easily be implemented to get a system of nonlinear algebraic equations that can be solved using Newton’s iterative method.

## 4. Convergence Analysis

Here, we will analyze the convergence of function approximation using the Laguerre wavelets by providing some theorems. Then, we introduce the absolute value between the exact and the approximate solutions obtained by the given method, after which we investigate the error of our approach. The following theorem shows that the function approximation based on the Laguerre wavelet converges to the function itself, and an upper bound is also obtained for an absolute value of series expansion coefficients.

**Theorem** **1.** *For a given continuous and bounded function f(x) on interval [0,1], the extension of the Laguerre wavelets, defined in Equation (8), converges uniformly to it.*

**Proof.**  Let $f\left(x\right)\in L^{2}\left[0,1\right]$ be a continuous function and |f(x)|≤R, where $R\in R^{+}$. Then, it can be assumed that f(x) is in the form of Equation (8). The coefficients of the Laguerre wavelet in the approximation of the function f(x) can be determined through the following relation:

$$c_{nm}=\langle \psi_{nm},f\left(x\right)\rangle .$$

Now, for n,m>0, we have

(15) $$c_{nm}=\langle \psi_{nm},f\left(x\right)\rangle =\int_{0}^{1}\psi_{nm}f\left(x\right)dx=\frac{2^{\frac{k}{2}}}{m!}\int_{I_{nk}}f\left(x\right)L_{m}\left(2^{k}x-2n+1\right)dx,$$

in which $I_{nk}=\left[\frac{n-1}{2^{k-1}},\frac{n}{2^{k-1}}\right)$. By assigning the variable $2^{k}x-2n+1=t$, we have

(16) $$c_{nm}=\frac{2^{\frac{-k}{2}}}{m!}\int_{-1}^{1}f\left(\frac{t-1+2n}{2^{k}}\right)L_{m}\left(t\right)dt.$$

Now, using the property of the Laguerre polynomials, we obtain

(17) $$|c_{nm}|\leq \frac{1}{2^{\frac{k}{2}}m!}\int_{-1}^{1}|f\left(\frac{t-1+2n}{2^{k}}\right)|\;|L_{m}\left(t\right)|dt\leq \frac{R}{2^{\frac{k}{2}}m!}\int_{-1}^{1}\left|L_{m}\left(t\right)\right|dt.$$

Since $L_{m}\left(.\right)$ is continuous and integrable on (−1,1), let $\int_{-1}^{1}\left|L_{m}\left(t\right)\right|dt=A$. Then, we have

(18) $$|c_{nm}|\leq \frac{RA}{2^{\frac{k}{2}}m!}.$$

This means that the series $\sum_{n=1}^{2^{k-1}}\sum_{m=0}^{M-1}c_{nm}$ is absolutely convergent; hence, the series presented in Equation (8) is uniformly convergent. □

A direct conclusion of this theorem is that the two-dimensional Laguerre wavelet expansion of function $f\left(x,y\right)\in L^{2}\left(\left[0,1\right]\times \left[0,1\right]\right)$, which is defined in Equation (9), also uniformly converges to it.

## 5. Illustrative Examples

In this section, we calculate the approximate solutions of the fractional Schrödinger Equation (1) based on the provided method by the Laguerre wavelets. We also use the following uniform norm to define the absolute error:

(19) $$E\left(x,y,t\right)=|U\left(x,y,t\right)-\tilde{U}\left(x,y,t\right)|,\;\left(x,y\right)\in \Omega ,\;t\in \left[0,1\right],$$

in which $\tilde{U}\left(x,y,t\right)$ introduces the approximation solution for problem (1) that was obtained by using the proposed method, while U(x,y,t) shows the exact solution. Our method is applied with $k_{1}=k_{2}=2$, $M_{1}=M_{2}=4$ and the number of equally central points distributed in the interval [0,1].

**Example** **1.** *For the first example, consider the following two-dimensional time–space fractional Schrödinger equation:*

(20) $$\partial_{t}^{\alpha }U+i\left(\partial_{x}^{\beta }U+\partial_{y}^{\beta }U\right)+iG\left(x,y,t\right)U=0,\;\left(x,y\right)\in \Omega ,\;t\in \left[0,1\right],\;i=\sqrt{-1},$$

*with the initial condition U(x,y,0)=sin(x+y), t∈[0,T], where $G\left(x,y,t\right)=\frac{3}{2}-2\frac{sin(x+y-0.5t)}{sin(x+y)}$. The exact solution of this problem was obtained for α=1 as follows [16,25]:*

$$U\left(x,y,t\right)=exp\left(\frac{-3it}{2}\right)sin\left(x+y\right).$$

**Figure 1:**
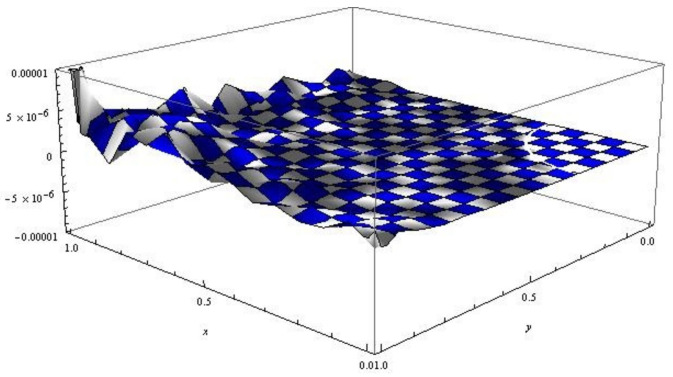
The error function for the numerical solutions of Example 1, with α=1 and β=1.2.

*The obtained approximate solutions from the Laguerre wavelet method are shown in Table 1 and Figure 1. It can be clearly seen in Table 1 that the numerical results of the proposed method with fewer repetitions achieved better accuracy from the reported results in [16].*

**Example** **2.** *In the second example, we further demonstrate the effectiveness of the proposed method. Consider Equation (1) with the initial condition $\phi \left(x,y\right)=x^{2}{\left(1-x\right)}^{2}y^{2}{\left(1-y\right)}^{2}e^{i}$ and $G\left(x,y,t\right)=1+\frac{1}{cos(\frac{\beta }{2}\pi )}\left[\vartheta \left(x,\frac{\beta }{2}\right)+\vartheta \left(y,\frac{\beta }{2}\right)\right]$, in which*

(21) $$\begin{array}{cc} \; & \vartheta \left(y,\frac{\beta }{2}\right)=\frac{1}{\Gamma (3-\beta )}\frac{y^{2-\beta }+{\left(1-y\right)}^{2-\beta }}{y^{2}{\left(1-y\right)}^{2}}-\frac{6}{\Gamma (4-\beta )}\frac{y^{3-\beta }+{\left(1-y\right)}^{3-\beta }}{y^{2}{\left(1-y\right)}^{2}}\\ \; & +\frac{12}{\Gamma (5-\beta )}\frac{y^{4-\beta }+{\left(1-y\right)}^{4-\beta }}{y^{2}{\left(1-y\right)}^{2}},\;\left(x,y\right)\in \Omega ,\;t\in \left[0,1\right]. \end{array}$$

**Figure 2:**
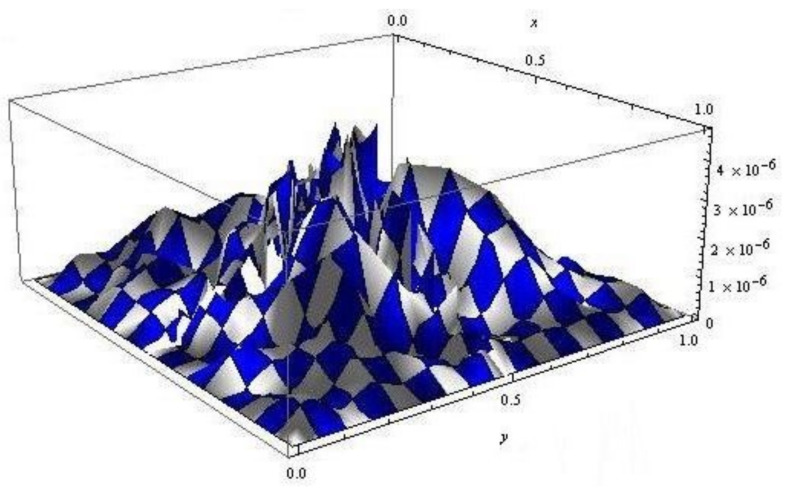
The error function for numerical solutions of Example 2, with α=1.

*We assume that $\kappa_{1}=\kappa_{2}=1$ in Equation (1). By choosing α=1, the exact solution of this problem is $U\left(x,y,t\right)=x^{2}{\left(1-x\right)}^{2}y^{2}{\left(1-y\right)}^{2}e^{i(t+1)}$ [15]. Table 2 shows the numerical errors of our method and the presented method in [15] for α=1 and different values of β. Figure 2 gives the associated error E(x,y,t) for β=0.85. The numerical results clearly show the improvement of accuracy by using the Laguerre wavelets approach. Therefore, our suggested method is more effective.*

## 6. Conclusions

In the present work, the Laguerre wavelets and the collocation method were employed to construct an original approach for solving 2-dimensional time–space fractional Schrödinger equations effectively. The treatment was found through a nonlinear discretization that made use of the Laguerre polynomials with wavelets and was compared with some examples taken from the literature of particular cases in which the fractional Schrödinger Equation (1) was solvable in explicit form. It was shown that the obtained results are in very good agreement with or even better than the previous methods. Moreover, we conclude that the proposed method is significantly more effective in solving this problem numerically.

## Figures and Tables

**Table 1:** Comparison of the absolute errors of the proposed method with different values for α,β in Equation (20).

Method	α	β=1.2	β=1.4	β=1.6	β=1.8	β=2
This study	α=0.0	$2.71578\times 10^{-4}$	$2.96775\times 10^{-4}$	$4.82481\times 10^{-5}$	$4.60978\times 10^{-6}$	$2.01762\times 10^{-6}$
$M_{1}=4$	α=0.2	$3.12299\times 10^{-4}$	$3.56969\times 10^{-4}$	$3.26856\times 10^{-4}$	$8.77692\times 10^{-5}$	$9.80176\times 10^{-7}$
$M_{2}=4$	α=0.4	$3.39405\times 10^{-5}$	$3.509808\times 10^{-5}$	$1.69322\times 10^{-5}$	$7.98751\times 10^{-6}$	$4.96747\times 10^{-6}$
	α=0.6	$2.013919\times 10^{-5}$	$1.52279\times 10^{-5}$	$3.83772\times 10^{-6}$	$3.74967\times 10^{-7}$	$2.17126\times 10^{-7}$
	α=0.8	$4.194561\times 10^{-6}$	$3.54682\times 10^{-6}$	$2.98667\times 10^{-6}$	$5.50806\times 10^{-7}$	$5.03257\times 10^{-7}$
	α=1.0	$2.35096\times 10^{-7}$	$2.34678\times 10^{-7}$	$1.90231\times 10^{-6}$	$2.11453\times 10^{-7}$	$1.60234\times 10^{-8}$
Method in [16]	α=1.0	$1.5595\times 10^{-5}$	$1.0943\times 10^{-6}$	$1.9856\times 10^{-5}$	$1.8697\times 10^{-6}$	$2.6566\times 10^{-7}$
with M=256						

**Table 2:** Comparison of the absolute errors of the proposed method for Equation (21).

Method	β=0.6	β=0.85
This study	$2.9073\times 10^{-6}$	$4.4034\times 10^{-6}$
Method in [15]	$2.19898\times 10^{-5}$	$4.7600\times 10^{-5}$

## Data Availability

Not applicable.
